# Pharmacological, antioxidant, genotoxic studies and modulation of rat splenocyte functions by *Cyperus rotundus* extracts

**DOI:** 10.1186/1472-6882-13-28

**Published:** 2013-02-06

**Authors:** Kilani-Jaziri Soumaya, Mhalla Dhekra, Châbane Fadwa, Ghedira Zied, Limem Ilef, Ghedira Kamel, Chekir-Ghedira Leila

**Affiliations:** 1 Laboratoire de Biologie Cellulaire et Moléculaire, Faculté de Médecine dentaire de Monastir, Université de Monastir, Rue Avicenne, Monastir, 5000, Tunisie; 2 Unité de Pharmacognosie/Biologie Moléculaire 99/UR/07-03. Faculté de Pharmacie de Monastir, Université de Monastir, Rue Avicenne, Monastir, 5000, Tunisie

**Keywords:** *Cyperus rotundus*, Analgesic activity, Anti-inflammatory activity, Lipid peroxidation effect, Chromosome aberrations, Immunomodulatory effect

## Abstract

**Background:**

*Cyperus rotundus* Linn. (Cyperaceae) is a Tunisian medicinal plant used in folkloric (traditional) medicine to treat stomach disorders and inflammatory diseases. The present study explored the analgesic, anti-inflammatory and genotoxic activities of extracts from the aerial parts of *C. rotundus*. The antioxidant capacity and the modulation of splenocyte functions by these extracts were also investigated in mice. The phytochemical analysis was carried out using standard methods.

**Methods:**

Aqueous, ethyl acetate, methanol and TOF-enriched extracts (300, 150, and 50 μg/ml) were evaluated for their analgesic and anti-inflammatory activities. 4, 2, and 1 mg/ml of each extract were tested to investigate their effect on lipid peroxidation. The genotoxic study was monitored by measuring the structural chromosome aberrations of mice treated with 300 mg/kg of extract. The proliferation of lymphocytes in the absence and presence of mitogens was assessed at a concentration range 1–1000 μg/ml.

**Results:**

The tested extracts were able to decrease the mouse ear oedema induced by xylene. Furthermore, it was shown that the same extracts reduced the number of abdominal contractions caused by acetic acid in mice, revealing the peripheral analgesic activity of these extracts. It is worth noting that mice treated with doses up to 300 mg/kg b.w. of *Cyperus rotundus* extracts did not exhibit any toxicity. The tested extracts significantly enhance lymphocyte proliferation at 1 mg/ml.

**Conclusions:**

It appears that *C. rotundus* extracts contain potent components such as flavonoids that may potentially be useful for modulating the immune cell functions, provoking analgesic, anti-inflammatory and antioxidant effects.

## Background

Tunisia is a rich source of medicinal plants, and a number of plant derived extracts are used to fight against various diseases. Only few plants have been scientifically explored. Plant derived natural products such as alkaloids, tannins, terpenes and flavonoids have received considerable attention in the recent years due to their diverse pharmacological properties including their analgesic, anti-inflammatory and antioxidant activities [1].

Pain is a pathophysiological response of the living tissue to undesirable stimuli. Inflammation can be defined as a reaction of a living cell or tissue to injury, infection or irritation/infiltration. It is characterized by swelling, redness and heat/fever. It can be induced by conditions that bring about the release of inflammatory mediators such as histamine, prostaglandins, nitric oxide, serotonin, cytokinines, leukotrienes, platelet activating factors and substance P [2]. In order to reduce the inflammatory response and damage to the human body, much effort has been made in the search for natural immunomodulatory products [3]. The pharmacology of pain has become a complex field. More recently, completely synthetic compounds based on natural pharmacophores have been introduced into the market but, research and medical fields still struggle with the side-effect profiles from these analgesic substances that are undesirable. Therefore, the development of newer and more substantial analgesic drugs with fewer side-effects is necessary.

*Cyperus rotundus* Linn, a sedge of the family of Cyperaceae, order cyperales, is widely distributed in the Mediterranean basin areas. This plant, which grows naturally in tropical, subtropical and temperate region, is widespread in the North-East, Center and South of Tunisia [4]. *C. rotundus* is a traditional medicinal plant appearing among the Indian, Chinese and Japanese natural drugs .It is used in the treatment of spasms, stomach disorder and inflammatory diseases [5-7]. Zhu et al. [8] described the ulcer inhibitory effect of decoction from the rizhomes of *C. rotundus*, related to the inhibition of gastric motility. Other pharmacological investigations have indicated that *C. rotundus*, has remarkable hypotensive [9] and antipyretic effects [10].

The aim of this study was to validate the phytochemical groups, the anti-inflammatory, analgesic and antioxidant effects of the different extracts from *Cyperus rotundus* aerial parts. Besides, we investigated the immunomodulatory activity of the same extracts on lymphocyte proliferation as well as their genotoxicity by measuring the chromosome aberrations in bone marrow cells.

## Method

### Plant material

*Cyperus rotundus* aerial parts were collected from the region of Monastir, located in the Center of Tunisia, in October 2008. The botanical identification was carried out by Prof. Med Chaieb (Department of Botany, Faculty of Sciences, University of Sfax, Tunisia), according to the flora of Tunisia [4]. A voucher specimen (Cp.10-08) is kept in the laboratory of Pharmacognosy, Faculty of Pharmacy of Monastir, for future references.

### Preparation of plant extracts

The fresh aerial parts of *Cyperus rotundus* were dried at room temperature and reduced to coarse powder. 100 g of the powdered leaves were boiled in water for 15 to 20 min. The crude extract obtained was filtered and lyophilized (aqueous extract). The residue was dissolved in water.

In order to obtain an extract enriched with Total Oligomer Flavonoids (TOF), 100 g of powder were macerated in water/acetone mixture (1v/2v) for 24 hours with continuous stirring. The extract was filtered and the acetone was evaporated under low pressure in order to obtain an aqueous phase. The tannins were partially removed by precipitation with an excess of NaCl for 24 hours at 5°C, then we recovered the supernatant. The latter was extracted with ethyl acetate, concentrated and precipitated with an excess of chloroform. The precipitate was separated and yielded TOF enriched extract which was dissolved in water.

Ethyl acetate and methanol extracts were obtained by soxhlet extraction (6 h). The two types of extracts, with different polarities, were concentrated to dryness and the residue was kept at 4°C. These two extracts were resuspended in DMSO.

### Quantitative analysis of extracts

#### Determination of total polyphenol and flavonoid content

The polyphenol content of *Cyperus rotundus* extracts was quantified by the Folin-Ciocalteau reagent [11,12]. Aliquots of test samples (100 μl) were mixed with 2.0 ml of 2% Na_2_CO_3_ and incubated at room temperature for 2 min. After the addition of 100 μl 50% Folin-Ciocalteau phenol reagent, the reaction tube was further incubated for 30 min at room temperature, and finally absorbance was read at 720 nm. Gallic acid (0.2 mg/ml) was used as a standard. A known volume of each extract was placed in a 10 ml volumetric flask to estimate flavonoid content according to the modified method of Zhishen et al. [13]. Distilled water was added to make the volume 5 ml, and then 0.3 ml NaNO_2_ aliquot (1:20 w/v) was added to this dilution. Three milliliters of AlCl_3_ (1:10 w/v) were added 5 min later. After a 6- min incubation, 2 ml of 1 N NaOH was added to the mixture and the total absorbance was measured at 510 nm [12]. Quercetin was used as standard for constructing a calibration curve.

#### Determination of tannin content

The method described by Pearson [14] was used for the determination of tannin content of the samples. The extraction of tannin in the sample was achieved by dissolving 5 g of the sample in 50 ml of distilled water in a conical flask, allowing the mixture to stand for 30 min with shaking of the flask at 10-min intervals, and then centrifuging at 5000 rpm for 15 min to obtain a supernatant (tannin extract). The extract was diluted to 100 ml in a standard flask using distilled water. Five milliliters of the diluted extract and 5 ml of standard tannic acid (0.1 g/L) were measured into different 50-ml volumetric flasks. One milliliter of Folin- Denis reagent was added to each flask, followed by 2.5 ml of saturated sodium carbonate solution. The solutions were made up to the 50-ml mark with distilled water and incubated at room temperature for 90 min [15]. The absorption of these solutions was measured against that of the reagent blank (containing only 5 ml of distilled water) in a Beckmann spectrophotometer at 760 nm wavelength. Tannin content (tannic acid equivalents) was calculated in triplicate and the concentration of tannin in the extract was determined using pure tannic acid as standard.

### Animals

Balb/c mice (6–8 week-old males, 20–30 g) were obtained from the Pasteur Institute (Tunis, Tunisia). The animals were housed in polypropylene cages with stainless steel grill tops and provided with bedding of clean paddy husk. The animals were acclimatized to laboratory conditions for one week prior to treatment. The temperature in the animal room was maintained between 25 ± 2°C with a relative humidity of 30–70% and illumination cycle set to 12 h light and 12 h dark. The mice were fed with standard laboratory pelleted feed. All animal experiments were performed in accordance with the guidelines for the care and use of laboratory animals published by the US National Institutes of Health. The study protocol was approved by the Ethics Committee of the University Hospital Fattouma-Bourguiba of Monastir, Tunisia.

### Acute toxicity

Mice were randomly divided into groups of 5 animals and treated by i.p. injection: a first control group was given solvent Tween-80 (1%) and a second control group was treated with Tween-80 (5%). In fact, aqueous, methanol and TOF-enriched extracts were dissolved in Tween-80 (1%), whereas ethyl acetate extract was dissolved in Tween-80 (5%). The other groups of animals were treated by different doses of extracts, ranging from 125 mg/kg to 1 g/kg body weights. The number of dead animals was followed every day for one week after treatment.

### Analgesic study: acetic acid-induced writhing assay

The analgesic effect was tested according to the method described by Shibata et al. [16]. The abdomen writhing is a model of visceral pain and it was produced by i.p. injection of 1% (v/v) acetic acid solution (volume of injection 0.1 ml/10 g b.w.) to each mouse, 30 min after the administration of the the aqueous, the TOF-enriched extract, ethyl acetate and methanol extracts (50, 150, 300 mg/kg, b.w, respectively). Tween-80 (1%, v/v) was used as negative control and diclofenac sodium was used as positive control (100 mg/kg, b.w.). After the immediate injection of acetic acid, each mouse was isolated in an individual observation box and the number of writhes produced in these animals was counted for 15 min.

The percentage analgesic activity was calculated as follows:

(1) $$\mathrm{Percentage}\;\mathrm{analgesic}\;\mathrm{activity}\;=\;\frac{{\mathrm{N-N}}^{t}\times \;100}{N}$$

Where N is the average number of stretching of control animals per group

And N^t^ is the average number of stretching of treated animals per group.

### Anti-inflammatory study: xylene-induced ear oedema

The mice were divided into groups of five. Thirty minutes after i.p. injection of the extract (50 to 300 mg/kg, b.w.) or dexamethasone (positive control) (300 mg/kg, b.w.), 0.03 ml of xylene was applied to the anterior and posterior surfaces of the right ear. The left ear was considered as control. Two hours after xylene application, the mice were sacrificed and both ears were removed. Circular sections were taken using a cork borer with a diameter of 7 mm, and were later weighed. The increase in weight caused by the irritant was measured by subtracting the weight of the untreated left ear section from that of the treated right ear sections. The oedema was quantified as the weight difference between the two earplugs. The anti-inflammatory activity was evaluated as percent oedema reduction/inhibition in the treated animals relative to the control animals [17,18] using the relation:

(2) $$\begin{array}{l} \mathrm{Oedema}\;\mathrm{reduction}\;\mathrm{inhibition}\;\%=100\;\times \;\left(\mathrm{Rt-Lt}\right)/\\ \;\left(\mathrm{Rc-Lc}\right) \end{array}$$

Where *R*t = mean weight of the right ear plug of the treated animals; *Lt* = mean weight of the left ear plug of the treated animals; *Rc* = mean weight of the right ear plug of the control animals; *Lc* = mean weight of the left earplug of the control animals.

### Evaluation of lipid peroxidation by the thiobarbituric acid (TBA) assay

TBA reacts with malondialdehyde (MDA) to form a diadduct, pink chromogen, which can be detected spectrophotometrically at 532 nm [19]. Normal male mice were used for the preparation of liver homogenate. The perfused liver was isolated, and 10% (w/v) homogenate was prepared with homogenizer at 0-4°C with 0.15 M KCl. The homogenate was centrifuged at 800 rpm for 15 min and clear cell-free supernatant was used for the study of *in vitro* lipid peroxidation. One ml of 0.15 M KCl, 0.1 ml of rat liver homogenate, 100 μl of ascorbic acid (0.5 mM) and 0.2 ml of each extract concentration (1 to 4 mg/ml) were mixed and the peroxidation reaction was initiated by adding 100 μl of 15 mM FeSO_4_. After the incubation at 37°C for 1 h, the reaction was stopped by adding 500 μl of trichloroacetic acid (TCA) (28%, w/v) and 380 μl TBA (2%, w/v). This final mixture was heated in a water bath for 20 min at 80°C, then it was cooled, centrifuged and the absorbance of the supernatant was measured at 532 nm. The inhibition percentage of lipid peroxidation was calculated by comparing the results of lipid peroxidation obtained with liver homogenates treated with FeSO_4_ and extracts to those of controls treated only with FeSO_4_, by using the following formula:

(3) $$\begin{array}{l} \mathrm{Inhibition}\;\mathrm{of}\;\mathrm{lipid}\;\mathrm{peroxidation}\left(\%\right)\\ \;=\left(\mathrm{Absorbance}\;\mathrm{control}-\mathrm{Absorbance}\;\mathrm{test}\right)/\\ \;\mathrm{Absorbance}\;\mathrm{control}\times 100. \end{array}$$

### Cell preparation from mice

Spleen Balb/c lymphocytes were obtained as previously reported [20]. Briefly, 8-week-old male Balb/c mice were killed by cervical dislocation. The spleen was isolated, aseptically removed and homogenized by mincing with a sterile forceps. The splenocytes were harvested by centrifugation (1500 rpm, for 10 min), and red blood cells were lysed with a lysis buffer (144 mM NH_4_Cl, 1.7 mM Tris Base). The cells were washed twice with phosphate-buffered saline (PBS), resuspended in complete RPMI medium and adjusted to 5×10^6^cell/ml. The cell incubation was conducted at 37°C, in 5% CO_2_ enriched atmosphere.

### Cell proliferation assay

The evaluation of lymphocyte proliferation was carried out by using the MTT [3-(4,5- dimethylthiazol-2-yl)-2,5-diphenyltetrazolium bromide] coloration method [21]. One hundred microliter of splenocyte suspension (3×10^6^ cells/ml) in a 96-well plate were preincubated in RPMI 1640 medium (Control) for 24 h before the addition of mitogens (LPS and lectin at 5 μg/ml), alone or with increasing concentrations of each extract (1, 0.1, 0.01, 0.001 mg/ml) solubilized in RPMI. The cells were incubated at 37°C in humidified 5% CO_2_ atmosphere for additionally 24 h. After centrifugation at 1500 rpm for 10 min, the cell pellet was re-suspended in 50 μl of MTT (5 mg/ml) in RPMI media and incubated for 4 h at 37°C in humidified 5% CO_2_ atmosphere. The culture medium was removed after centrifugation and dimethylsulfoxide was added. The absorbance was measured at 570 nm.

The percentage of proliferation was calculated by the following equation:

(4) $$\begin{array}{l} \mathrm{Proliferation}\left(\%\right)=\left((\mathrm{OD}\;\mathrm{sample-OD}\;\mathrm{control}\right)/\\ \;\left(\mathrm{OD}\;\mathrm{control})\right)\times \;100 \end{array}$$

### Chromosome aberrations assay

The aim of this experiment was to investigate if *C. rotundus* extracts exhibit a genotoxic effect, using animal models. Blab/c mice were used (average body weight 20 ± 0.3 g; age: 6 weeks old). These mice were given a standard granulated food and drinking water, and were divided into four groups with 5 mice each, as follows:

Group 1: Mice given Tween-80 (1%).

Group 2: Mice given Tween-80 (5%).

Group 3: Mice given 40 mg/kg b.w. of methyl methane sulfonate (MMS), as positive control.

Group 4: Mice given 300 mg/kg b.w. of *Cyperus rotundus* extracts.

The animals within the different treatment groups were given a subcutaneous injection of yeast suspension, dissolved in water (20 mg/ml), to accelerate the mitosis of bone marrow cells [22]. Ten minutes later, they received a single intraperitoneal (I/P) injection of the tested substance solution (200 μl of MMS, *Cyperus rotundus* extracts or Tween-80). Twenty-four hours later, the different animals were sacrificed by cervical dislocation. 200 μl of vinblastin (250 μg/ml) were injected (I/P injection) to animals 45 minutes before they were sacrificed in order to block the dividing cells in metaphase. The cells of the bone marrow were collected from femurs and tibias, shielded with a hypotonic shock solution (KCl 0.075 M), then harvested in the presence of a methanol–acetic acid mixture 3/1 (v/v) (3 repetitions) by centrifugation at 1200 rpm for 10 minutes, according to the technique described by Evans et al. [23]. The cells were spread on glass slides that were blazed on a flame for 5 s, then air-dried for conservation at room temperature and finally stained with a 4% (v/v) Giemsa solution in water, for 15 min [24]. After coding slides, the chromosomes of 100 cells blocked in metaphase were examined for chromosome abnormalities at a magnification of 100 × using an optical microscope (Olympus, France). Three replicates (300 metaphases per dose level) for each controls and treated groups were conducted. The chromosome aberrations were identified according to the criteria described by Savage [25]. The metaphases with chromosome breaks, gaps, rings and centric fusions were recorded and expressed as percentage of total metaphases per group.

### Statistical analysis

Data are expressed as mean ± SD (Standard Deviation). The biological activity was examined in three individual experiments, performed in triplicate for each dose. The statistical significance was determined using the Student’s t-test. P<0.05 was considered as indicative of significance as compared to the control group.

## Results

### Chemical preliminary study and metabolite content evaluation of C*yperus rotundus* extracts

The chemical preliminary study of *Cyperus rotundus* extracts showed the presence of flavonoids, tannins and polyphenols. The Folin-Ciocalteu method is a rapid and widely-used assay investigating the total phenolic content, but it is known that different phenolic compounds gave different responses with this method [26]. Therefore, we expressed the total polyphenol content of the extracts as gallic acid equivalents [27] following confirmation of linearity of the response of the assay using the extracts. The total flavonoid content of the *C. rotundus* extracts was determined by the method of Zhishen et al. [13]. The results summarized in Table 1 reveal that TOF-enriched extract exhibited the most important flavonoid and total polyphenolic contents, followed by methanolic, aqueous and ethyl acetate extracts. The aqueous extract showed the highest extracting yield (13.7%), whereas the TOF enriched extract showed the lowest one (0.16%) (Table 1).

**Table 1:** **Quantitative polyphenol, flavonoid, and tannin contents of extracts from *C. rotundus *aerial parts**

**Metabolites**	**Extracts**			
	**TOF enriched extract**	**Methanolic extract**	**Aqueous extract**	**Ethyl acetate extract**
**Yields (%, w/w)**	**0.16**	**13.12**	**13.7**	**1.52**
**Polyphenols (gallic acid equivalents)**^**a**^	**700 ± 56**	**355 ± 7**	**220 ± 28**	**175 ± 21**
**Flavonoids(quercetin equivalents)**^**a**^	**1610 ± 77**	**320 ± 4**	**316±15**	**315 ± 14**
**Tannins (tannic acid equivalents)**^**a**^	**46,25 ± 0**	**62,86 ± 6**	**82,51 ± 8**	**200,84 ± 2**

a: means of three experiments.

It appears that 1 mg of TOF-enriched extract contains an amount of polyphenols equivalent to 700 μg of gallic acid and an amount of flavonoids equivalent to 1610 μg of quercetin. However, 1 mg of each of ethyl acetate, methanolic and aqueous extracts contains respectively up to 175, 355 and 220 μg equivalent of gallic acid and 315, 320 and 316 μg equivalent of quercetin. The highest content of tannin was recorded in both ethyl acetate and aqueous extracts, with respectively, 200.84 μg and 82.51 μg equivalent of tannic acid. Yet, the methanolic and TOF-enriched extracts tannin contents were respectively, equivalent to 62.86 and 46.25 μg of tannic acid.

### Acetic acid-induced writhing assay

When administered (I/P) to mice, the different doses of the extracts (50, 150 and 300 mg/kg) provoke reduction of writhing response induced by acetic acid administered intraperitonally to mice (Table 2), in a dose-dependent manner. The inhibition percentages of writhing obtained with aqueous, ethyl acetate and methanolic extracts were 65.7%, 63% and 43%, respectively at the same dose (300 mg/kg, b.w.). The TOF-enriched extract showed the most significant inhibitiory effect (92.8% at the dose of 300 mg/kg b. w.). The standard analgesic diclofenac sodium (100 mg/kg, b.w.) reduced the abdominal constriction to 100%.

**Table 2:** **Analgesic effect of the different extracts of *Cyperus rotundus *on acetic acid-induced writhings**

**Treatment**	**Doses (mg/kg)**	**Acetic acid writhing contraction**^**a**^	**Percent inhibition (%)**
**Control**	**-**	14 ± 0,70	**-**
**Diclofenac sodium**	**25**	10,33 ± 4,04	**26,21**
	**50**	1,5 ± 0,7*	**89,30**
	**100**	0 ± 0*	**100**
**TOF enriched extract**	**50**	5,4 ± 0,54*	**61,42**
	**150**	3,2 ± 0,83*	**77 ,2**
	**300**	1 ± 1*	**92,8**
**Aqueous extract**	**50**	11 ± 1,58*	**21,5**
	**150**	8 ± 0,70*	**42,9**
	**300**	4,8 ± 0,83*	**65,7**
**Ethyl acetate extract**	**50**	10 ± 2,73*	**28,6**
	**150**	8,2 ± 1,92*	**41,4**
	**300**	5,2 ± 1,30*	**63**
**Methanolic extract**	**50**	8 ± 2,88	**10**
	**150**	11,2 ± 1,92*	**20**
	**300**	8 ± 1*	**43**

^a^ Values are expressed as mean ± standard deviation (*n* = 5 animals). *P < 0,05 compared to corresponding control (Student’s *t*-test).

### Xylene-induced mouse ear oedema assay

The aqueous, ethyl acetate, methanolic and TOF-enriched extracts from *Cyperus rotundus* administered at the dose of 300 mg/kg, b.w showed a significant inhibiting effect against ear oedema induced by xylene. The inhibition percentages were 74.38%, 62.73%, 44.6% and 77.25%, respectively, while the positive control, dexamethasone (300 mg/kg, b.w.) exhibited an inhibition percentage of ear oedema of 68.81% as shown in Table 3.

**Table 3:** **Effect of the extracts from *Cyperus rotundus *on xylene-induced ear edema in mice**

**Treatment**	**Doses (mg/kg)**	**Edema (mg)**^**a**^	**Inhibition (%)**
**Control**	**-**	61,12 ± 7,44	**-**
**Dexamethasone**	**50**	49,46 ± 1,95*	**19,08**
	**150**	37,9 ± 2,57*	**38,48**
	**300**	19,06 ± 1,61*	**68,81**
**TOF enriched extract**	**50**	37,46 ± 4,7*	**38,71**
	**150**	25,38 ± 3,35*	**58,47**
	**300**	13,9 ± 1,79*	**77,25**
**Aqueous extract**	**50**	42,52 ± 2,72*	**30,43**
	**150**	28,9 ± 2,01*	**52,71**
	**300**	15,66 ± 1,05*	**74,38**
**Ethyl acetate extract**	**50**	35,26 ± 1,97*	**40,77**
	**150**	30,24 ± 1,33*	**50,52**
	**300**	22,78±1,19*	**62,73**
**Methanolic extract**	**50**	50,76 ± 3,48*	**16,95**
	**150**	44,98 ± 3,05*	**26,41**
	**300**	33,86 ± 2,10*	**44,6**

^a^ Values are expressed as mean ± standard deviation (*n* = 5 animals). *P < 0,05 compared to corresponding control (Student’s *t*-test).

### Antioxidant activity

**Figure 1:**
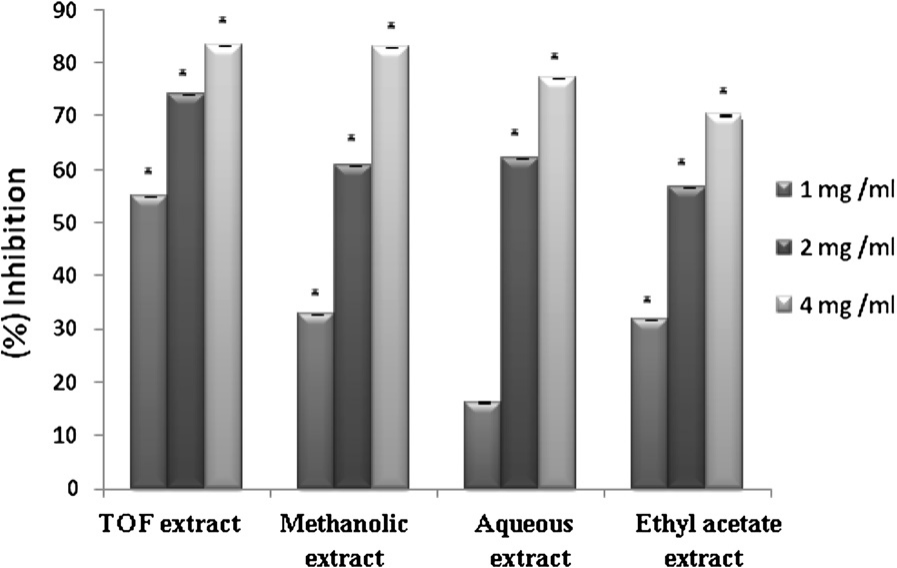
**Anti-lipid peroxidation activity of different extracts from *Cyperus rotundus *.** Data represented as mean ± standard error of three replicated experiment. Values in each line with asterisks (*) are significantly different (P<0.05).

The antioxidant activity of the plant extracts was evaluated by quantifying the ability of the different extract concentrations to suppress iron (Fe^2+^)-induced lipid peroxidation (LPO) in rat liver homogenate. In fact, the addition of FeSO_4_ in liver slices resulted in enhancing lipid peroxidation as observed earlier by other workers [28]. The initiation of LPO by FeSO_4_ is known to take place through ferryl perferryl complex [29]. The assessment of the extent of lipid peroxidation relied on individual determinations of MDA contents in sample supernatants by performing the TBARS assay, as described by Draper and Hadley [30]. MDA is an end product of peroxidative decomposition of polyenoic fatty acids by the lipid peroxidation process. Its accumulation in tissues is indicative of the extent of lipid peroxidation [30]. *Ex vivo* experiments showed that different concentrations (1, 2, 4 mg/ml) of each extract produced dose-depending inhibitory effects on lipid peroxidation induced in rat liver cells (Figure 1). IC_50_ values of MDA formation were 1.3 mg/ml in the presence of the aqueous extract, 1.2 mg/ml in the presence of the methanolic extract and 1.7 mg/ml in the presence of the ethyl acetate extract. The TOF-enriched extract (IC_50_= 0.75 mg/ml) exhibited the most significant lipid peroxidation inhibitory effect as compared to the others extracts. Thus, it is notable that lipid peroxidation (LPO) inhibition capacity of the extracts increases as the extract concentrations increase.

### Acute toxicity testing

The different extracts of *Cyperus rotundus* did not exhibit acute toxicity up to the dose of 1 g/kg b.w. The weight of the treated mice varied normally after 7 days of observation. The common side effects such as mild diarrhea, loss of weight and depression were not recorded. In fact, the mice treated with doses up to 300 mg/kg b.w. of *C. rotundus* extracts exhibited significant anti-inflammatory and analgesic activities. For this reason, we attempted to investigate whether this dose would provoke any genotoxic effect.

### Genotoxicity evaluation using the chromosome aberration assay

The metaphasic chromosome slides were observed for structural aberrations. The observations revealed the presence of centric fusions, rings and chromosomal breaks (Table 4). The positive control (MMS) was found to induce statistically significant number of structural aberrations, mainly centric fusions, rings and chromosome breaks in bone marrow cells. The treatment of animals with the different extracts at 300 mg/kg b.w. for 24 h did not induce significant number of chromosome aberrations when compared to the negative control. These data indicate that *C. rotundus* extracts exhibit no genotoxic activity, and thus support the safety of *C. rotundus* extracts at the tested dose.

**Table 4:** **Different types of chromosomal damage induced by MMS (40 mg/kg b. w.) and different extracts (300 mg/kg b.w.) from *Cyperus rotundus *in bone marrow cells of male Balb/c mice**

**Treatment**	**ctb**	**csb**	**del**	**r**	**cf**	**ra**	**Total aberrations**^**a**^
**Solvent control : Tween-80 (1%)**	**-**	**1**	**-**	**6**	**6**	**-**	**13 ± 0.58**
**Methyl methansulfonate (MMS)**	**-**	**16**	**-**	**17**	**12**	**-**	**44 ± 3.06**
**TOF enriched extract**	**-**	**3**	**-**	**3**	**3**	**-**	**9 ± 1.53**
**Aqueous extract**	**-**	**4**	**-**	**4**	**2**	**-**	**10 ± 0.62**
**Methanolic extract**	**-**	**-**	**-**	**3**	**11**	**-**	**14 ± 0,58**
**Solvent control : Tween-80 (5%)**	**-**	**-**	**-**	**10**	**7**	**-**	**17 ± 1.15**
**Ethyl acetate extract**	**-**	**2**	**-**	**12**	**5**	**-**	**19 ± 1.53**

ctb = chromatid break, csb = chromosome break, del = deletion, r = ring, cf = centric fusion, ra = rearrangements.

^a^Total aberrations (mean ± standard error of the mean from one hundred cells were analyzed per animal for a total of 500 cells per treatment).

### Mitogen-induced splenocyte proliferation

**Figure 2:**
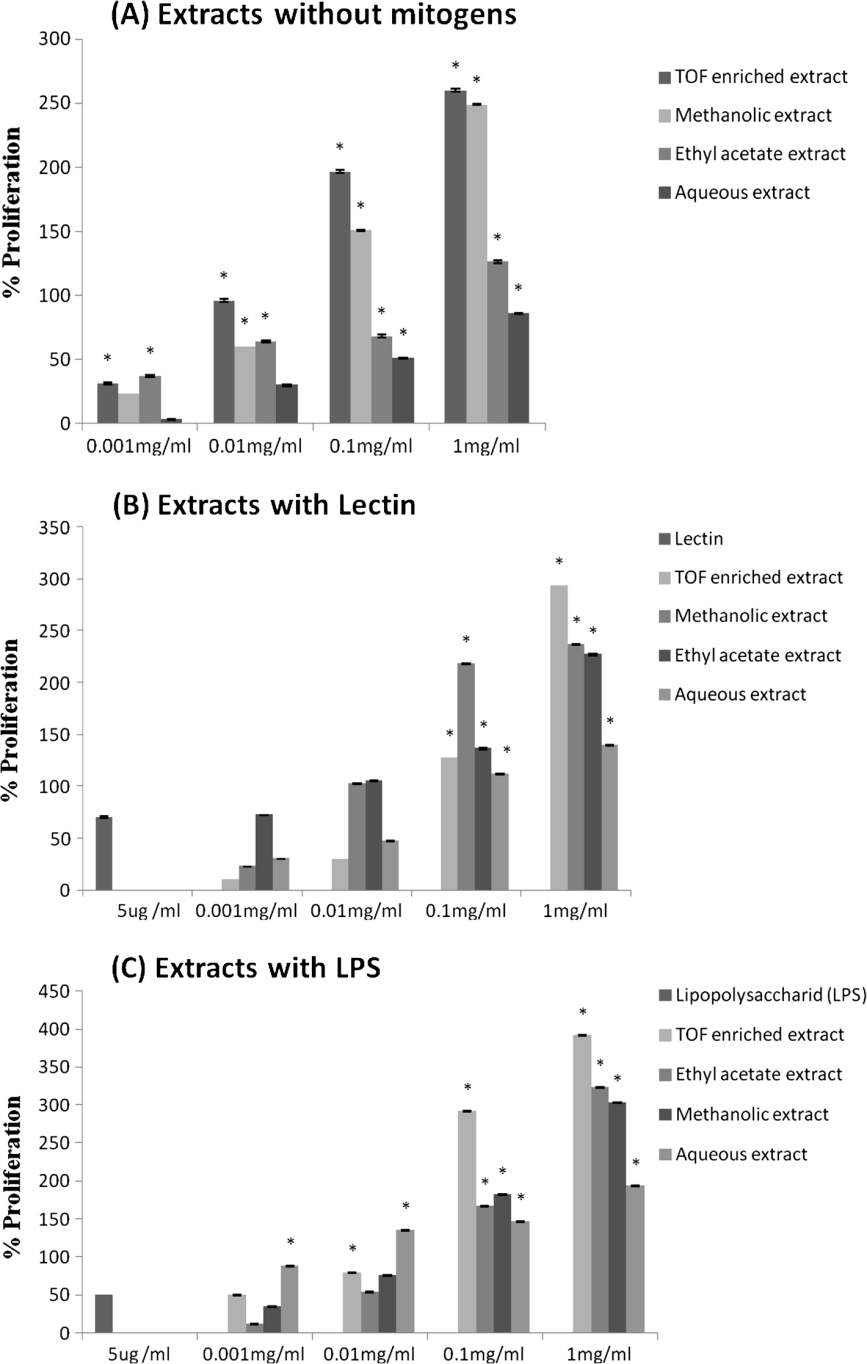
**Effects of different extracts from *Cyperus rotundus *on *in vitro *proliferation response of splenocytes: (A) Cells were incubated with extract without mitogens, (B) cells were treated with extracts in the presence of 5 μg/ml lectin, and (C) Cells were treated with extracts in the presence of 5 μg/ml LPS.** Cell proliferation was assessed by the MTT test. The data plotted represent average ± standard deviation of triplicates. Values in each line with asterisks (*) are significantly different (P<0.05).

The lymphocyte-mediated immunity plays an important role in the cellular and humoral immune responses. The capacity to elicit an effective T- and B-lymphocyte immunity can be shown by the stimulation of lymphocyte proliferation response. It is generally known that Lectin stimulates T cells and LPS stimulates B cell proliferation [31,32]. In this study, we investigated the effects of the aerial part extracts from *C. rotundus*, on lymphocyte proliferation with and without mitogen stimulation. In the absence of mitogens, all the dose-dependently extracts stimulate splenocyte proliferation compared to untreated cells. As shown in Figure 2-A, maximums of cell proliferation were observed at a concentration of 1 mg/ml of aqueous extract (86.06%), ethyl acetate extract (126.22%), methanolic extract (249%) and TOF-enriched extract (260.11%). When we treated the mouse splenocytes with mitogen, we also observed a stimulation of cell proliferation. A dose of 5 μg/ml LPS stimulates cell proliferation by 50.82%, whereas a dose of 5 μg/ml of lectin stimulates proliferation by 69.67%. In the presence of lectin, the different extracts elicited an increase in splenocyte proliferation by 139.8%, 227%, 236.88% and 293.44% at 1 mg/ml, respectively (Figure 2-B), compared to the untreated cells. In the presence of LPS, the extracts at 1 mg/ml enhanced the proliferation of splenocytes by 193.44%, 323.7%, 303.28% and 391.8% with respectively the same extracts (Figure 2-C), compared to the untreated cells. A dose of 1 mg/ml of each extract exhibits no toxicicity on mouse splenocytes as it was revealed by MTT assay (survival rates higher than 90%).

## Discussion

Previous studies conducted by our team revealed the presence of catechin, afzelechin and galloyl quinic acid in TOF-enriched extract from the aerial parts of *C. rotundus*, and luteolin, ferulic acid, quercetin, 3-hydroxy, 4-methoxybenzoic acid and 6,7 dimethoxycoumarin in the ethyl acetate extract [33]. Among these secondary compounds, belonging to either tannin, polyphenol or flavonoid families, several are known to exhibit antinociceptive and anti-inflammatory effects [34,35], and we believe that the analgesic and anti-inflammatory activities we observed with extracts from the aerial parts of *Cyperus rotundus* should be ascribed to such compounds*.* Besides*,* i.p. administration of different doses (125 mg/kg to 1 g/kg b.w.) of *Cyperus rotundus* extracts to mice gives no toxic effect. This finding suggests that the plant extracts are, at the tested doses, safe in mice.

The peripheral analgesic effect may be mediated through the inhibition of cyclo-oxygenases and/or lipoxygenases (and other inflammatory mediators) [36]. This hypothesis is in accordance with those of Adeyemi et al. [37] and Zhang et al. [38], who have postulated that acetic acid-induced writhing is a highly sensitive and useful test for analgesic drug development, especially peripherally acting analgesics. Acetic acid induces pain by liberating endogenous substances (bradykinin, serotonin, histamine, substance P) [39], which in turn excite the pain nerve endings. In our observation, aqueous, methanolic, ethyl acetate and TOF-enriched extracts significantly (P < 0.001) reduced the abdominal constriction response induced by the acetic acid in a dose-dependent manner. TOF-enriched extract exhibited the most analgesic potency, whereas methanolic extract showed the weakest one. This result could be explained by the presence of active compounds diluted and/or masked by various components in the methanolic extract and which are more accessible and/or more concentrated in the TOF- enriched extract. Besides, this test is useful for the evaluation of mild analgesic non steroidal, anti-inflammatory compounds [40,41]. This suggests a peripherally induced mechanism of the analgesic action for *Cyperus rotundus* extracts [42]. Therefore, one possible mechanism of the analgesic activity by *C. rotundus* extracts could be due to the blockage of the effect, or the release of endogenous substances (arachidonic acid metabolites) that excite pain nerve endings by the pharmacologically active principles of *C. rotundus* extracts. From a mechanistic point of view, the lack of specificity in acetic acid-induced writhing test suggests the involvement of different nociceptive mechanisms in the reduction of muscular constriction, such as sympathetic system, through the release of biogenic amines, cyclooxygenases and their metabolites inhibition, and through opioids receptors mechanisms [43].

In evaluating the anti-inflammatory effect, it is important to estimate the activities of the extracts in the acute phase as well as in the chronic phase of inflammation. Xylene-induced ear oedema in mice was selected to evaluate acute anti-inflammatory activity and is a good *in vivo* test useful for evaluating lipoxygenase inhibitors and partially associated with substance P [44]. The results in Table 3 showed that the different extracts of *C. rotundus* caused significant inhibition of oedema as compared to the control group. These results may suggest that the plant extracts exert significant anti-inflammatory activity, especially in the acute inflammatory response. The suppression of this response is a likely indication of the antiphlogistic effect [45]. The topical anti-inflammatory effect suggests that constituents of the extract may relieve rheumatism and offer the additional advantage of suppressing inflammatory response initiated by tissue injury when the leaves are used in folkloric treatment of arthritis and wounds respectively [46]. In fact, the activity may be related to the inhibition of inflammatory mediators such as histamine, serotonin, bradykinin, and prostaglandins.

On the other hand, the anti-inflammatory and analgesic activities of many plant extracts have been attributed to their high sterol/triterpene [47] or flavonoid contents [34]. Other studies have demonstrated that various flavonoids such as rutin, quercetin, luteolin, hesperidin and biflavonoids produced significant antinociceptive and/or anti-inflammatory activities [48]. On the basis of these results, it can be concluded that the different extracts of *C. rotundus* possess antinociceptive and anti-inflammatory activities and this may be due to any one or a combination of their phytochemical constituents [33].

The difference in significance between ethyl acetate and TOF-enriched extracts concerning the analgesic and anti-inflammatory activities may be due to their different constituents. Several compounds obtained from *C. rotundus* extracts were identified as catechin, luteolin, quercetin and ferulic acid [33]. Previous reports indicated that various flavonoids have an anti-inflammatory activity [48] suggesting that flavonoids should be responsible for the anti-inflammatory and analgesic effects obtained with the different *C. rotundus* tested extracts. In addition, we previously showed that ethyl acetate extract contains sterols [49], which are documented as anti-inflammatory constituents [50,51].

Several medicinal plants are considered immunomodulatory as they display a variety of anti-inflammatory, antimicrobial and antitumoral effects [20]. The modulation of immune cell activities by molecules from medicinal plant origin is an area of active interest for inflammation, autoimmunity and cancer therapy. Investigating the effects of substances that promote or inhibit lymphocyte inflammatory response represents a potent means to study immunomodulation and drug discovery. In the present study, lymphocytes isolated from mice were used as models because of their critical role in controlling innate and immune inflammatory responses [52]. Lymphocytes are the key effector cells of mammalian adaptive immune system and our studies show that the different subpopulations of lymphocytes are differentially activated by *Cyperus rotundus* extracts at varying levels. The extracts significantly increased the proliferation of splenocytes at concentrations of 1–1000 μg/ml, and these effects were markedly enhanced in the presence of LPS. The concomitant presence of lectin and extracts (1 mg/ml) enhance splenocyte proliferation, except with the methanolic extract. Lectin acts directly on lymphocyte T cells, which are involved in cell mediated immunity, while LPS acts on lymphocyte B cells that are responsible for humoral immune response [20]. Therefore, *C. rotundus* extracts could enhance both cellular and humoral immunity depending on the concentrations. A dose of 1 mg/ml stimulates cellular and humoral immunity. In fact, lymphocyte B cell proliferation as well as lymphocyte T cell response are stimulated by both high and low extract concentrations. We believe that phenolic compounds, especially flavonoids, might contribute to the immunomodulatory effects of *C. rotundus* extracts and their anti-inflammatory activity.

Injury of the mucous membranes cells may provoke the production of active oxygen species. The latter can directly injure the surrounding cells and extracellular matrices, such as hyaluronic acid, and produce lipid peroxides and metabolites of arachidonic acid. In general, active oxygen species are thought to promote inflammation through these processes [53]. On the other hand, antioxidants, such as superoxide dismutase and catechins, are known to suppress inflammation. Antioxidants may offer resistance against the oxidative stress by scavenging the free radicals, inhibiting the lipid peroxidation, and/or by other mechanisms, and thus preventing some diseases [54]. The different extracts of *C. rotundus* exhibited important antioxidant activities. The results obtained in the present study may be attributed to several reasons *viz*: the inhibition of ferryl-perferyyl complex formation; scavenging of OH or superoxide radicals or by changing the ratio of Fe^3+^ / Fe^2+^; reducing the conversion rate of ferrous to ferric iron or by chelating of the iron itself. The antioxidant activity of the extracts may probably be due to the rapid and extensive degradation of the oxidant principles in an *ex vivo* state. In our study, there seems to be a good correlation between the phenolic content and antioxidant activity of the extracts, since the TOF-enriched extract with higher phenolic content showed the highest antioxidant activity. However, it is known that nonphenolic antioxidants could also contribute to the antioxidant activity of an extract. Thus, we strongly believe that the anti-inflammatory activity revealed by *C. rotundus* plant extracts, should be ascribed, at least in part, to their antioxidant activity as far as radical-induced actions play a central role in the inflammatory condition. Thus, the antioxidant and free radical scavenging flavonoids could prevent generation of inflammatory mediators [55].

Based on these results, we chose to perform the genotoxicity assay on mice by numbering the chromosome aberrations in sample treated animal cells, and comparing them to control animal cells. The chromosomal aberrations (CA) are changes in chromosome structure resulting from a break or exchange of chromosomal material. Most of the CA observed in cells are lethal, but there are many aberrations that are viable and can cause genetic effects, either somatic or inherited [56]. Our study showed no induction of chromosome or chromatid aberrations in the cells obtained from animals treated with each tested extract. To explain this result, we propose the following hypotheses: [1] mutagenic compounds were deactivated in the animal body, [2] mutagenic compounds did not reach bone marrow cells, [3]*C. rotundus* extracts does not cause genetic damage at all, or does not cause genetic damage detected by using the chromosome aberrations assay.

## Conclusions

The results of the present work clearly demonstrate the significant anti-inflammatory and peripheral analgesic activities of the different extracts from the aerial parts of *Cyperus rotundus*. The same extracts exhibited an anti-lipid peroxidation capacity. On the other hand, the tested extracts influence humoral-mediated immunity by stimulating B and T cell proliferation. These results indicate that *Cyperus rotundus* might have immunostimulatory properties that can be exploited in food and pharmaceutical industries. The analgesic, anti-inflammatory, antioxidant and immunomodulatory effects of the extracts may be ascribed to their flavonoid, tannin and polyphenol contents. The exact mechanism and the bioactive principles responsible for these actions still need to be explained. However, further *in vivo* studies are needed to evaluate the real use of these extracts in the anti-inflammatory and antioxidant therapeutics.

## Competing interests

The authors declare that they have no competing interests.

## Authors’ contributions

KJS carried out polyphenols, flavonoids and tannins quantification, photochemical analysis, extract preparation and chromosome aberrations assay. MD carried out analgesic assay. CF carried out anti-inflammatory assay. GZ carried out lipid peroxidation assay. LI prepared solutions which have been used for splenocyte proliferation assay. GK assisted with Cyperus rotundus extraction and study design and interpretation. CGL helped conceive the study and helped in the preparation of the manuscript. All authors read and approved the final manuscript.

## Pre-publication history

The pre-publication history for this paper can be accessed here:

http://www.biomedcentral.com/1472-6882/13/28/prepub
